# Cultivating Professional Identity Through Community‐Based Dental Education: A Qualitative Study

**DOI:** 10.1002/jdd.13947

**Published:** 2025-06-01

**Authors:** Sruthi Sunil, Nidhi Gupta, Kamran Ali, Trine Fink, Xiangyun Du

**Affiliations:** ^1^ College of Dental Medicine QU Health Qatar University Doha Qatar; ^2^ Department of Sustainability and Planning Aalborg UNESCO PBL Centre Aalborg University Aalborg Denmark; ^3^ Department of Health Science and Technology Aalborg University Aalborg Denmark

**Keywords:** community‐based dental education (CBDE), dental curriculum, predoctoral dental education, professional identity (PI), sociocultural approach

## Abstract

**Aim:**

This study aimed to explore the role of community‐based dental education (CBDE) in shaping professional identity (PI) among predoctoral dental education students at a newly established dental college.

**Materials and Methods:**

Qualitative methods employing focus groups were used for data collection. A purposive sampling technique was used to recruit participants meeting predefined eligibility criteria. Separate focus groups were planned for each cohort, and the aim was to recruit 6–10 participants for each focus group. The study adopted a subject‐centered sociocultural approach and thematic analysis to explore participants’ experiences highlighting how CBDE may contribute to PI development in predoctoral dental education.

**Results:**

A total of 19 predoctoral dental students participated in three separate focus groups, comprising six students from Year 3, seven students from Year 4 and six students from Year 5. Deductive thematic analysis mapped CBDE activities across the four PI development dimensions: personal, clinical, interpersonal, and cultural. In the personal dimension, participants emphasized the role of self‐confidence and self‐reflection in their professional growth. The clinical dimension highlighted the development of decision‐making and technical skills through direct patient interaction. The interpersonal dimension underscored the importance of mentorship and teamwork in improving communication and problem‐solving skills. Exposure to diverse patients enhanced cultural competence, thus enabling tailored and sensitive care. CBDE supported all dimensions, facilitating PI development in predoctoral dental education.

**Conclusion:**

CBDE plays a vital role in shaping PI by enhancing confidence, clinical expertise, decision‐making, communication, and cultural competence. These experiences prepared students for the complexities of contemporary dental practice and diverse healthcare environments. Despite the benefits of CBDE, challenges such as close supervision, balancing outreach programs with academic demands, and addressing resource limitations in diverse settings were noted.

**Abbreviations**
CBDE: community‐based dental education
IRR: inter‐rater reliability
PBL: problem/project‐based learning,
PI: professional identity

## 1 Introduction

Professional identity (PI) refers to “how we perceive ourselves within our occupational context and communicate this to others” [1]. PI is a multifaceted concept studied for over 60 years, with psychological perspectives focusing on self‐concept, values, and beliefs, while sociological views emphasize social interactions and cultural norms. PI may enhance personal satisfaction in fulfilling expected roles as a professional [1, 2]. In healthcare, PI construction involves students developing attitudes, beliefs, and standards that align with their responsibilities as future healthcare professionals [2]. PI development is also influenced by the educational environment, mentorship, role models, and self‐reflection [3]. There is growing interest in fostering PI development in predoctoral (undergraduate) healthcare programs. Previous research in healthcare education has also identified professional attributes such as cultural competence, social responsibility, empathy, and accountability in patient care for PI development [2]. PI development has been explored in healthcare professions and has been primarily focused on enhancing the clinical competence of students [4]. However, PI is a broader concept and should encompass skills in communication, teamwork, and collaborative practices in workplace settings [5].

Community‐based dental education (CBDE) represents a structured learning approach that allows students to pursue academic goals and provide oral health services to the community simultaneously. It promotes active engagement with the community and emphasizes reflective practices [6]. Accreditation guidelines emphasize the importance of providing opportunities for students to participate in service‐based learning and community‐based learning experiences within dental education programs [7, 8]. Several studies have explored CBDE's global uptake as an educational model by providing students with valuable experience in cultural competence, collaboration, practice management, while addressing oral health needs in underserved communities and delivering financial benefits to schools [7, 9]. However, challenges such as treating anxious patients, language barriers, and building trust can limit students' confidence in community settings [10].

Over the past few decades, PI development has been supported through diverse pedagogical approaches such as, problem/project‐based learning (PBL), case‐based learning (CBL), team‐based learning (TBL), and reflective practices [11]. However, the literature on PI in dental education shows limited understanding of how predoctoral dental students utilize their learning experiences to develop PI in community settings [12]. Although previous studies have demonstrated that social interactions in multiple learning settings may influence PI development, the association between PI and CBDE remains underexplored [13]. To address these gaps, this study aimed to investigate how predoctoral dental students perceive their PI development within CBDE, thereby focusing on the following research question: “How do dental students anticipate their PI development, and what factors contribute to its development?” The objectives were to explore the perceptions and experiences of predoctoral students regarding PI formation in CBDE and to identify the factors that influence PI development of dental students in community settings.

### 1.1 Conceptual Framework of PI Formation in CBDE

CBDE has become a vital component of contemporary dental education [14]. This study provides a framework to link CBDE and PI with four interrelated dimensions:

(1) *Personal dimension*: CBDE may help in the development of student agency by strengthening their self‐confidence, motivation, self‐image, and career choices through mentorship, teamwork, and real‐world patient interactions; it also encourages self‐directed learning and professional accountability, thereby positively influencing PI [15, 16, 17].

(2) *Clinical dimension*: CBDE may provide opportunities to consolidate clinical skills, time management, decision‐making, record‐keeping, and specialist referral to facilitate professional growth of learners [5].

(3) *Interpersonal dimension*: CBDE facilitates teamwork, mentorship, and collaborative skills among students through engagement with their peers, faculty, and the community. Learning activities such as situational analysis and project‐based assignments cultivate individual and collective agency, which are vital for PI development [17, 18].

(4) *Sociocultural dimension*: CBDE provides opportunities for students to consider the social, cultural, linguistic, and ethnic background of patients to provide personalized clinical care [19, 20, 21].

## 2 Materials and Methods

### 2.1 Ethics Approval

Ethical approval was obtained by the institutional review board of Qatar University (IRB No. QU‐IRB 1877–EA/23 dated‐ March 27, 2024) and Aalborg University, Denmark (IRB No. AAU084‐1074902, dated August 5, 2024).

### 2.2 Study Design

A qualitative research design using focus groups was considered to be most appropriate to answer the research question and objectives of the current study. Qualitative methods allow for an in‐depth exploration of participants'’ views and experiences to gain a deeper understanding of the multifaceted dimensions of PI development [4].

### 2.3 Study Setting and Duration

This study was conducted at a newly established College of Dental Medicine in Qatar. Data collection for this study was conducted from September 10 to November 20, 2024.

### 2.4 Sampling Technique and Participants

A purposive sampling technique was used to recruit participants meeting predefined eligibility criteria summarized as follows:

Eligibility criteria are as follows:

- Predoctoral dental students enrolled in the Doctor of Dental Medicine (DDM) program
- Students who had completed one or more courses in CBDE.

Exclusion criteria are as follows:

- Students from earlier years or those with no exposure to CBDE
- Students who had interrupted their studies

### 2.5 Sample Size

One focus group was planned for each cohort in Year 3, Year 4, and Year 5 with an aim to recruit 6–10 participants in each focus group in line with existing guidelines on sample size for qualitative focus groups [22]. Separate focus groups were planned based on their year of study to account for potential variations in the experiences of individual cohorts.

### 2.6 Data Collection

An open invitation to participate was sent to the 72 senior‐cohort students who met the eligibility criteria through the college's Student Affairs Office, which also acted as gatekeeper. The invitations were sent by email and were accompanied by a participant information sheet, which explained the purpose and scope of the study and included a list of frequently asked questions (FAQs). The rights of the participants were clearly explained including the voluntary nature of participation and a right to withdraw from the study. The contact details of the principal investigator (S.S.) were provided to the participants if they had any questions or needed any clarifications.

A topic guide was developed, informed by the literature and guided by the theoretical framework, to align with the objectives of the study. Mutually convenient date and time of each focus group was planned by the research team by liaising with students who responded to the invitations. Due to the clinical placements of Year 4 and Year 5 participants at off‐campus sites, focus groups for these cohorts were conducted online using Microsoft Teams. This platform was selected for its user‐friendliness, accessibility, and ability to facilitate interactive discussions in a secure environment [23].

All participants were required to submit a signed copy of a consent form to the prior to attending a focus group. At the commencement of each focus group, participants were reminded of the Chatham House rule [24]. The professional background and role of the moderator as a member of the dental faculty were explained to the participants of each focus group for transparency. Participants were reassured that all data would be treated confidentially and they were encouraged to express their views freely and also interact with each other. All focus groups were moderated by the principal investigator (S.S.) who had previous training and experience in qualitative research [25]. Each focus group was conducted in English and lasted 50–60 min. All focus groups were audio‐recorded with prior agreement of all the participants. Supplementary hand notes were also taken by the moderator.

### 2.7 Data Analysis

All focus group recordings were transcribed verbatim. Thematic analysis, integrating deductive and inductive approaches, were used to connect the theoretical foundation and empirical data of this study. The initial coding framework was formed by pre‐existing literature and research objectives, allowing for the identification of themes that aligned with the theoretical framework. This was followed by an inductive approach to further explore subthemes and categories, as well as new, unexpected themes that may emerge from the data without being constrained by pre‐existing assumptions. This ensured that the analysis captured participants’ perspectives and experiences beyond the predefined framework.

The content analysis focused on condensing meanings relevant to the context, with the research team remaining mindful of their emic status to ensure that the participants' voices and cultural context guided the interpretation [26]. Data charting was conducted using Microsoft Excel, and a codebook was developed. Coding of the complex qualitative data based on PI themes was carried out using NVIVO 14 software (Lumivero, Denver, CO, USA) to extract the key data items [27]. The four themes in the study's conceptual framework served as the initial basis for categorizing the data. Multiple rounds of transcript reviews were conducted to ensure the meanings were accurately categorized and aligned with the context. The emerging themes were identified and collated under each dimension of the framework, which was compared within each group and among separate groups [28].

To ensure the credibility and trustworthiness of the study, all focus groups were moderated by the same researcher, and a second reviewer acted as a co‐coder for the transcripts, thus applying a thematic analysis approach combined with open coding [11]. The reviewer was provided with the codebook for data extraction, and this auditing process contributed to the validity of the analysis. Inter‐rater reliability (IRR) was assessed in 10% of the focus group interviews, yielding a reliability score of 90%. Each coder independently extracted data into the codebook, with the second coder blinded to the first author's data to minimize bias. Member checking was performed by inviting an independent qualitative researcher to review the thematic analysis. Minor enhancements to the thematic analysis were made following member checking. Feedback was also sought from five participants to verify that the data interpretations reflected their views. The researchers’ emic position was recognized during data analysis, and reflexivity was maintained through self‐reflection and maintaining personal diaries.

## 3 Results

A total of 19 predoctoral dental students participated in three separate focus groups, comprising six students from Year 3 (*n* = 6), seven students from Year 4 (*n* = 7) and six students from Year 5 (*n* = 6). Thematic analysis of the data was contextualized to the four dimensions of PI, which informed the conceptual framework of the study as summarized in Table 1. Each dimension includes underlying factors that may influence PI development in CBDE.

**TABLE 1:** Summary of thematic analysis with key themes and subthemes.

Themes	Subthemes
Personal dimension	Self‐confidence
	Self‐reflection
	Motivation
	Career perspectives
Clinical dimension	Decision‐making skills
	Technical skills
Interpersonal dimension	Mentorship
	Team working skills
	Communication skills
	Collaboration with peers, supervisors, and organizations
Socio‐cultural dimension	Socio‐cultural awareness
	Strategies to cope with cultural diversity

The results of thematic analysis related to each dimension of the conceptual framework of the study are explained below and show how they may contribute to PI development in CBDE. Each section is supported by verbatim quotes by the participants.

### 3.1 Personal Dimension

Thematic analysis within the personal dimension identified factors that may facilitate their PI development by the participants, namely, self‐confidence, self‐image, reflection, and motivation.

#### 3.1.1 Development of Self‐Confidence

A dominant theme across all focus groups was the significant role of CBDE in improving self‐confidence. More than half of the participants across all years emphasized how their experiences in CBDE activities contributed to their confidence in their journey to become dentists. They associated this confidence with exposure to real‐world practice, including delivering oral health education to diverse populations. As some participants mentioned,

The more exposure we get, the more confident we become. (Group 3, Student 2)

It boosted my confidence when we moved to year four, like the clinical phase, because we experienced treating real patients in primary care. (Group 2, Student 7)

#### 3.1.2 Career Perspectives

A subset of participants, predominantly from senior cohorts, noted that CBDE activities transformed their self‐image, thereby enabling them to feel more like dentists. These experiences allowed them to link the learning activities in CBDE with their career goals and professional growth.

Community interactions are very important for us, as dentists know how to deliver information and educate patients. Even though we do not specialize in public health, it's a part of our career. (Group 3, Student 5)

#### 3.1.3 Self‐Reflection

Self‐reflection emerged as a key subtheme reported by some participants (*n* = 5 in both Group 1 and Group 3). Reflective practices in CBDE allowed participants to evaluate their actions, decision‐making, and interactions, thus enabling personal and professional growth. This process encouraged accountability, helping them refine skills, recognize areas for improvement, and understand their impact on patient outcomes and the healthcare team. As a result, they play a crucial role in shaping their development as thoughtful, empathetic, and competent healthcare providers [29].

Self‐reflection is something that I feel is very important. It helps me understand how I can improve as a student. (Group 1, Student 1)

#### 3.1.4 Learner Motivation

Few participants (*n* = 5) reported feeling motivated by the patient‐centered nature of CBDE, which allowed them to see the direct impact of their work on underserved populations. Participants felt motivated by interacting with real patients, deepening their commitment to the profession, and appreciating tangible outcomes such as improved patient understanding and better preventive care compliance [13, 21].

### 3.2 Clinical Dimension

The participants across all the focus groups agreed that CBDE facilitated the development of clinical *decision‐making* and *technical skills*. They widely agreed that the structured learning environment, combined with real‐world exposure, boosted their confidence in making informed clinical decisions. These experiences sharpened their analytical thinking and reinforced the importance of integrating theoretical knowledge with practical application [21, 30–32].

The participants (*n* = 3) emphasized that frequent practice and direct patient interaction allowed them to refine their technical abilities. They highlighted that the focus on real‐life scenarios not only enhanced their proficiency but also deepened their understanding of patient needs and the nuances of treatment protocols. Approximately half of the participants across all years noted that CBDE was instrumental in bridging the gap between theoretical knowledge and clinical practice, thus enabling them to provide patient‐specific care [16]. The opportunity to engage in supervised, practical sessions was often described as a critical component of their learning journey, thus bridging the gap between theoretical knowledge and clinical practice [33, 34].

You can apply skills you've learned on real patients,…and I do not think you can learn this from textbooks. (Group 3, Student 4)

CBDE enabled participants to tailor oral health education to diverse patient needs and develop critical decision‐making skills. They learned to adapt plans based on the available resources and feedback, boosting their confidence in making well‐rounded clinical decisions [32, 35].

We get opportunities to consider treatments that are in the best interest of individual patients, and this learning experience is very valuable. (Group 2, Student 2)

### 3.3 Interpersonal Dimension

#### 3.3.1 Role of Mentorship in Enhancing Learning and Communication Skills

Participants across all focus groups emphasized the positive impact of mentorship from supervisors, dental assistants, and nurses in primary care settings. This support not only enhanced their learning and confidence but also facilitated the development of their communication skills. By actively seeking help and feedback from faculty and staff, participants improved their ability to collaborate and convey their needs effectively, thus highlighting the interpersonal dimension of their growth [36, 37].

My supervisor helped me understand the unique treatment needs of different patients we encounter in primary care settings. (Group 3, Student 1)

The nurses and assistants at the primary healthcare center were invaluable…always ready to help and provide whatever support we needed. (Group 2, Student 3)

#### 3.3.2 Building Team Working Skills

Communication with colleagues and team members was highlighted as a critical factor in learning. The participants (*n* = 8) from all three focus groups expressed their appreciation for opportunities to engage with peers, mentors, and patients. These interactions were instrumental in enhancing their problem‐solving abilities and strengthening teamwork skills, which are crucial for their professional development [31].

I have developed crucial team‐building skills with clear and effective communication, which has strengthened my collaboration with the entire team. (Student 6, Group 3)

It was easier for us because the supervisors and nurses understood that it was our first time, and they provided a supportive and collaborative team environment. (Student 2, Group 3)

#### 3.3.3 Development of Awareness Through Patient Communication

Participants valued CBDE for improving patient communication, thereby facilitating patient‐centered care and building rapport. Direct engagement enhanced empathy, adaptability, and clarity, thus shaping them into compassionate providers. The participants highlighted the importance of effective communication with patients, emphasizing how it contributed to the development of their problem‐solving skills and ability to address patient needs more holistically [13, 21].

Communication skills with patients and the team, I feel is most important in our career. (Student 1, Group 2)

I learned how to communicate with people from diverse backgrounds. (Student 3, Group 3)

#### 3.3.4 Collaboration/Site Partnership

A few participants (*n* = 4) from the senior cohort highlighted that CBDE extends beyond providing care to patients from lower socioeconomic backgrounds. They emphasized the importance of collaborating with higher authorities involved in policy development. This exposure allowed them to understand the broader systemic factors influencing oral healthcare delivery and the role of advocacy in shaping policies to improve community health outcomes [33, 38].

More networking opportunities within the field of public health. (Student 5, Group 3)

### 3.4 Sociocultural Dimension

The participants recognized the cultural dimension of CBDE as an essential aspect of their professional growth. They developed a deeper sensitivity to cultural differences through direct interactions with diverse patient populations and learned to tailor their care to meet varying cultural needs. This exposure helped them enhance their cultural competence, ensuring that they could provide respectful, effective care to individuals from all backgrounds. The experience helped the participants to appreciate the importance of cultural awareness in building trust and rapport with patients [39, 40].

#### 3.4.1 Socio‐Cultural Awareness

Through CBDE, all the participants gained exposure to diverse populations, helping them adapt to various cultural and clinical needs. This exposure also encouraged a deeper appreciation of cultural sensitivities in patient care. Cultural awareness, respect for differences, and adapting communication styles to different cultural needs emerged as important elements in the development of their PI [32, 36].

It gives us exposure to different population groups within the community. (Group 2, Student 6)

We gained valuable exposure to diverse individuals in the community, which allowed us to view their oral health through a lens we had not considered before…. (Group 2, Student 2)

#### 3.4.2 Development of Strategies to Cope With Cultural Differences

The participants reported the importance of treating diverse populations, including those with special needs, which required them to adapt their clinical approach and communication strategies. Furthermore, they recognized that cultural differences such as language barriers, varying health beliefs, and differing attitudes toward dental care could influence patient cooperation and treatment outcomes. The participants learned to actively listen to patients’ concerns, respect their cultural practices, and adjust treatment approaches to be more culturally appropriate. By developing these skills, they were able to build stronger, more trusting relationships with patients, thereby ensuring that they received the best possible care while respecting their unique cultural contexts.

It taught me the importance to treat everyone equally and appreciate the differences in attitudes toward dental treatment. (Group 3, Student 2)

It is about knowing the environment and the treatment needs of the community—how you communicate with them and earn their trust. (Group 3, Student 6)

### 3.5 Challenges and Barriers

The participants reported challenges in maintaining motivation, particularly when patients showed resistance to giving up deleterious habits such as smoking or when patients seemed disinterested in recommendations for dental treatment.

To change patients’ existing habits can be difficult sometimes. (Group 3, Student 4)

Some participants in the senior cohort (*n* = 2) emphasized the need for more theoretical knowledge for better clinical application to improve their clinical decision‐making.

Having a stronger foundation in theory would make a big difference in how we approach patient care, empowering us to make more informed and effective clinical decisions…. (Group 3, Student 5)

This feedback highlights the need for stronger integration of theoretical understanding with hands‐on practice, which could enhance their preparedness for clinical challenges. In addition, some participants found it challenging to provide effective advice when patients had short attention spans, thus leading to brief interactions and reduced engagement with preventive care advice.

It may be more practical to limit the oral health advice to a short duration because people, especially younger ones, may have a limited attention span. (Group 3, Student 6)

A key challenge noted by several participants was communicating effectively with patients from diverse linguistic and cultural backgrounds. Language barriers, especially with patients who did not speak English or Arabic, made it difficult to convey information clearly. One participant described:

“The language barrier at first, especially with workers who couldn't speak English or Arabic, made it hard to deliver information.” (Group 3, Student 1).

If there is a language barrier, it can be very challenging…. (Group 3, Student 4)

In addition to language barriers, cultural norms such as gender preferences for dentists complicate patient care. For example, some female patients preferred to be treated by female dentists, which presented a barrier for male participants.

“Some females, particularly, prefer not to be treated by male dentists” (Group 2, Student 1), which was agreed upon by few other participants (*n* = 3).

Another significant obstacle was patient noncompliance with preventive care advice. Many participants highlighted the difficulty in convincing patients to prioritize dental health outside of emergency situations.

Convincing patients to prioritize their dental health beyond emergencies or pain was a challenge. (Group 3, Student 3)

These challenges highlight the need for culturally competent communication strategies to overcome both linguistic and cultural barriers and improve patient adherence to treatment plans.

## 4 Discussion

The findings of this study highlight the complex and multifaceted nature of PI formation in CBDE. Through thematic analysis, four crucial dimensions influencing students’ PI development were identified that illustrate the intricate nature of CBDE, which extends beyond enhancing clinical proficiency to inculcate self‐reflection, effective communication, and cultural sensitivity in community settings. It also underscores the transformative approach of CBDE on students’ professional growth, and how exposure to real‐world settings enhances learning and social accountability. This key aspect in healthcare education can be obtained through community engagement and collaborations [41, 42, 43]. This process facilitates their gradual transition from peripheral participation to active membership, thus emphasizing the importance of participation and social interaction in professional learning. Students develop a broader understanding of social accountability as healthcare providers through engagement with diverse patient populations and community needs [44, 45]. This holistic approach equips them to navigate the environment in contemporary dental practice, address health disparities, and provide culturally competent care. Although social accountability did not emerge as a distinct theme, it relates to the interpersonal and socio‐cultural dimensions of PI. The findings also highlight the critical role of self‐confidence and motivation in PI development, with most students reporting increased confidence due to hands‐on experience in community settings, aligning with literature on the link between experiential learning and enhanced clinical confidence [46, 47]. The theme of self‐image development aligns with Cruess et al. (2014) model of PI formation, which emphasizes education, mentorship, and experiential learning.[48] CBDE supports this model by exposing students to real‐life patient encounters to mold their professional behaviors and attitudes [48]. While many participants noted the transformative role of CBDE in shaping their self‐image and career aspirations, responses to motivation were mixed [49]. Some found the activities motivating, whereas others felt demotivated by resistant patients. This highlights the emotional complexities of CBDE, where self‐reflection practices are essential for managing challenges and aligning personal and professional expectations [50]. In addition, participants valued clinical exposure for advancing their skills, particularly in primary care settings, demonstrating how experiential learning bridges the gap between theoretical knowledge and real‐world application [47, 51].

The focus on patient safety and individualized treatment reflects participants’ internalization of the core values of patient‐centered care [52]. Participants emphasized the importance of mentors, supervisors, and team members in facilitating a supportive learning environment, essential for PI development. Communication with supervisors and peers was crucial in enhancing problem‐solving skills in real‐world patient care [53]. However, participants also noted challenges in communicating with patients from diverse cultural and linguistic backgrounds, particularly language barriers while conveying oral health advice to non‐native speakers. This highlights the need for enhanced training in cross‐cultural communication. In addition, CBDE exposes students to diverse populations, yet challenges such as cultural preferences for dentists and the promotion of preventive care highlight the need for enhanced cultural sensitivity training and practical strategies to address cultural dynamics in patient care [54].

In summary, this study shows CBDE may serve as a transformative pedagogical tool that not only equips students with essential clinical competencies but also plays a pivotal role in shaping their PI. The participants of the current study viewed their PI development as a dynamic and evolving process influenced by structured experiences, real‐world exposure, and mentorship. Engagement with diverse patient populations, participation in community‐based care contributed to their professional growth. Students highlighted key factors that may shape their PI, including application of scientific knowledge in real‐life contexts, mentorship modeling professional behavior, and appreciating cultural diversity to provide personalized clinical care. Confidence‐building, cultural awareness, and reflective practices enabled critical self‐evaluations and the alignment of personal and professional goals. However, the path to becoming dentist may present challenges such as balancing academic and clinical demands, processing feedback, and managing uncertainty during the transition from learners to practitioners.

## 5 Practical Implications

This study contributes to the growing body of literature on CBDE by offering a nuanced understanding of how it shapes PI. Several recommendations can be made to improve the CBDE experience and enhance PI development: enhancing reflective practices by incorporating structured reflection opportunities throughout the CBDE curriculum to encourage continuous self‐assessment and allowing students to track their growth, challenges, and emotional responses to patient care. Integrating cultural competence through theoretical knowledge on the cultural challenges in CBDE, communication, and patient diversity can better prepare students for inclusive practice. Strengthening mentorship by providing guidance and combining theory with hands‐on experience will enhance student's clinical decision‐making and competency development. By addressing these recommendations, CBDE programs can further enhance the PI development of dental students, thus equipping them with the confidence, competence, and cultural sensitivity required for their future careers.

## 6 Limitations and Future Perspectives

This study has several limitations. First, the data were derived from a limited sample size, which may not be fully representative of the diverse experiences of all dental students. Furthermore, as a single‐institution study conducted in a country with a unique sociocultural context, the findings may not be generalizable to institutions in other countries. Focus group discussions may have introduced social desirability bias, with students potentially sharing views they believed were expected of them rather than reflecting their genuine experiences. Although the researchers’ positionality is acknowledged, potential influence on participants’' behaviors and reflections cannot be ruled out. As the current study was conducted at a single institution, a comparison group of students without exposure to CBDE could not be included. This is acknowledged as a limitation and may be addressed in future studies. Future research can focus on conducting studies with larger, more diverse samples across various institutions and cultural contexts to improve generalizability. The research should specifically focus on the cultural aspects of PI development especially, how local values, traditions, and social interactions influence students’ experiences in CBDE. The use of mixed methods research to triangulate qualitative findings with quantitative data may provide a more comprehensive understanding on the impact of CBDE. Subsequent research in this direction could unravel the complex interplay between CBDE and PI development.

## 7 Conclusion

CBDE may support PI development in predoctoral dental education by bridging the gap between education and practice, thus enhancing students’ clinical preparedness, communication skills, and professionalism. In this study, participants reported positive experiences with CBDE, highlighting its role in cultivating PI across multiple dimensions to navigate the complexities of modern dental practice. The findings also identified some challenges in the delivery of CBDE that need to be addressed to further enhance the learning experiences of the students.

## Author Contributions

Sruthi Sunil, Xiangyun Du, and Kamran Ali designed the study and significantly contributed to the pedagogical conceptualization of the paper. Sruthi Sunil conducted most focus groups, analyzed the data, and wrote the initial manuscript draft. Nidhi Gupta served as a second independent coder. All authors reviewed, edited, and approved the final manuscript.

## Ethics Statement

Ethics approval was obtained from Qatar University (IRB No. QU‐IRB 1877–EA/23) and from Aalborg University (IRB no. AAU084‐1074902) and written consent was obtained from the study participants.

## Consent

No written consent has been obtained from the patients as there is no patient identifiable data included.

## Conflicts of Interest

The authors declare no conflicts of interest.

## Data Availability

All data generated or analyzed during this study are included in this manuscript, and the underlying data are available to the corresponding author upon request.
